# Cytotoxicity, inflammation, biomineralization, and immunoexpression of IL-1β and TNF-α promoted by a new bioceramic cement

**DOI:** 10.1590/1678-7757-2020-0033

**Published:** 2020-08-05

**Authors:** Leopoldo COSME-SILVA, Amanda Ferreira dos SANTOS, Camila Soares LOPES, Renan DAL-FABBRO, Francine BENETTI, João Eduardo GOMES-FILHO, India Olinta de Azevedo QUEIROZ, Edilson ERVOLINO, Naiana Viana VIOLA

**Affiliations:** 1 Universidade Federal de Alagoas Faculdade de Odontologia Departamento de Endodontia MaceióAlagoas Brasil Universidade Federal de Alagoas (UFAL), Faculdade de Odontologia, Departamento de Endodontia, Maceió, Alagoas, Brasil.; 2 Universidade Federal de Alfenas Faculdade de Odontologia Departamento de Clínica e Cirurgia AlfenasMinas Gerais Brasil Universidade Federal de Alfenas (UNIFAL), Faculdade de Odontologia, Departamento de Clínica e Cirurgia, Alfenas, Minas Gerais, Brasil.; 3 Universidade Estadual Paulista Faculdade de Odontologia Departamento de Endodontia AraçatubaSão Paulo Brasil Universidade Estadual Paulista (UNESP), Faculdade de Odontologia, Departamento de Endodontia, Araçatuba, São Paulo, Brasil.; 4 Universidade Federal de Minas Gerais Faculdade de Odontologia Departamento de Odontologia Restauradora Belo HorizonteMinas Gerais Brasil Universidade Federal de Minas Gerais (UFMG), Faculdade de Odontologia, Departamento de Odontologia Restauradora, Belo Horizonte, Minas Gerais, Brasil.

**Keywords:** Biocompatibility, Biomaterials, Cytotoxicity, Pulpotomy, Dental Pulp

## Abstract

**Aim:**

To evaluate the cytotoxicity, biocompatibility and mineralization capacity of BIO-C PULPO, and MTA.

**Methodology:**

L929 fibroblasts were cultured and MTT assay was used to determine the material cytotoxicity on 6, 24, and 48 h. A total of 30 male rats (Wistar) aged between 4 and 6 months, weighing between 250 and 300 g were used. Polyethylene tubes containing BIO-C PULPO, MTA, and empty tubes were implanted into dorsal connective tissue. After the experimental periods (7, 15, 30, 60, and 90 days) the tubes were histologically analyzed using hematoxylin-eosin (H&E), immunolabeling of IL-1β and TNF-α, and von Kossa staining, or without staining for polarized light analysis. The average number of inflammatory cells was quantified; the mineralization assessment was determined by the area marked in μm2 and semiquantitative immunolabeling analyses of IL-1β and TNF-α were performed. Then, data underwent statistical analysis with a 5% significance level.

**Results:**

It was observed that BIO-C PULPO and MTA presented cytocompatibility at 6, 24, and 48 similar or higher than control for all evaluated period. On periods 7 and 15 days, BIO-C PULPO was the material with the highest number of inflammatory cells (p<0.05). On periods 30, 60, and 90 days, BIO-C PULPO and MTA presented similar inflammatory reactions (p>0.05). No statistical differences were found between Control, BIO-C PULPO, and MTA for immunolabeling of IL-1β and TNF-α in the different periods of analysis (p<0.05). Positive von Kossa staining and birefringent structures under polarized light were observed in all analyzed periods in contact with both materials, but larger mineralization area was found with BIO-C PULPO on day 90 (p<0.05).

**Conclusion:**

BIO-C PULPO was biocompatible and induced mineralization similar to MTA.

## Introduction

Pulpotomy is the treatment for reversible pulpal lesions and it can be performed on permanent or deciduous teeth. After removal of the coronary pulp, a material must be chosen for insertion in the pulp region and this is a relevant factor that influences successful treatment.^1-3^ This material should be biocompatible, bactericidal, harmless to tissue, and promote healing of the pulp tissue.^4,5^ Furthermore, conservative treatment of the dental pulp has the advantage of preserving the vital function of the pulp, including the defensive mechanism and proprioceptive function of the teeth.^2^

Some materials have already been used in pulpotomy, such as Formocresol (FC),^3^ Calcium Hydroxide (CH),^4^ Ferric Sulfate (FS),^3^ Mineral Trioxide Aggregate (MTA),^6-8^ and Biodentine (Biodentine; Septodont Inc., Saint-Maur-des-Fosses, France).^6-7^ MTA is considered the gold standard for pulpotomies and other clinical applications for its physical-chemical and biological properties.^1,2,5-9^ MTA is a biocompatible material, that presents hydrophilic properties enabling its use even in the presence of moisture.^7^ Biological properties of calcium silicate-based cements, such as MTA, are related to the release of calcium, since calcium stimulates the proliferation of cells present in pulp tissue besides of mineralization. The stimulus to mineralization is relevant, as it allows the deposit of a mineralized tissue barrier on the pulp surface.^10^ Moreover, this mineral has appropriate radiopacity, low solubility, high pH, and antimicrobial activity.^8^

Despite the advantages, MTA has some disadvantages such as difficult handling,^11^ granular consistency,^12^ discoloration of dental crown,^13^ and slow setting time.^12^New materials based on calcium silicate are being designed for pulpotomy, as Bioceramic (BIO-C PULPO) (Angelus Industry, Londrina, Brazil).

BIO-C PULPO is bioceramic material composed of calcium silicate [tricalcium silicate and dicalcium silicate], calcium aluminate, calcium hydroxide, zirconium oxide, calcium fluoride, silicon dioxide, and iron oxide in the powder part and distilled water, plasticizing material, calcium chloride, and methylparaben, in the liquid part. According to the producer, besides being a material for pulpotomy, BIO-C PULPO is also indicated for cavity preparation and basis for restoration. Once the available literature lacks papers evaluating the biological properties of BIO-C PULPO, this study aimed to evaluate the cytotoxicity, inflammatory tissue response, mineralization capacity, and immunolabeling of interleukin (IL)-1β and tumor necrosis factor (TNA)-α of this material comparing gto white MTA-Ang (Angelus Industry, Londrina, Brazil).

## Methodology

### *In Vitro* Study

L929 fibroblast line cells were grown in Dulbecco modified Eagle medium (DMEM) supplemented with 10% fetal bovine serum (FBS) (Gibco BRL, Gaithersburg, MD), streptomycin (50 g/mL), and 1% antibiotic/antimycotic cocktail (300 U/mL, 300 mg/mL streptomycin, 5 mg/mL amphotericin B) (Gibco BRL) under standard cell culture conditions (37º C, 100% humidity, 95% air, and 5% CO_2_).^14^

The white MTA-Ang and BIO-C PULPO materials were prepared according to the manufacturer’s recommendations: for white MTA-Ang, 1 spoon of powder with 1 drop of distilled water, and for BIO-C PULPO, 1 spoon of powder with 3 drops of liquid.

### Preparing of the extract

The preparing of material extracts was performed according to previous study and also according to ISO 10993-5:2009.^15,16^ Disks containing these materials were prepared under aseptic conditions by using a sterile cylindrical polyethylene tube (diameter, 5 mm; height, 3 mm). Disks were kept in a 5% CO_2_ incubator at 37º C for 6 h for final setting.^14,16^ Then, disks with materials were removed from the mold and sterilized with ultraviolet light for 1 h.^14^ Each disk was immersed into 1 mL DMEM with 10% FBS and incubated in a humidified atmosphere containing 5% CO_2_ for 24 hours, according to ISO 10993-5:2009.^15^ Then, disks were discarded, and the supernatants (eluate extract) were collected and filtered through a sterile 0.22-mm filter (Sigma-Aldrich, St Louis, MO), to remove any suspended particles from the materials in the extracts.^14,17^ L929 fibroblasts were seeded into the 96-well plates (10^4^ cells/ well) and incubated for 24 h in a humidified air atmosphere of 5% CO_2_ at 37º C to enable cell attachment. The undiluted extract (1:1)^16^ was used for 6, 24, and 48 h. The L929 fibroblasts cell cultured in medium without extract served as the control. The MTT (3-(4,5-dimethylthiazol-2- yl)-2,5-diphenyltetrazolium bromide) solution (Sigma-Aldrich) was added to the cells, and fibroblasts were incubated at 37º C for 4 h protected from light to determine cell viability. MTT solution was discarded, and 200 mL isopropyl alcohol was added to each well. The plate was maintained under continuous stirring for 30 min to dissolve the dark blue crystals. The blue solution was transferred to a 96-well plate to measure the optical density at 570 nm in a spectrophotometer. The experiments were performed in triplicate.^16^

### *In Vivo* Study

The Research Ethics Committee of the School of Dentistry, Federal University of Alfenas (UNIFAL-MG) approved the study (protocol No. 692/2015). Authors followed the ARRIVE (Animal Research: Reporting of *In Vivo* Experiments) guidelines. Thirty male rats (Wistar) aged between four and six months, weighing between 250-300g were used. Sample size was estimated based on data from a previous study^18^ but using 90% power sample, which is corroborated by previous studies^19,20^and considering alpha error of 0.05 to recognize a significant difference, a minimum number of six rats/ analysis period was necessary. The rats were maintained in cages with three animals each, with free access to food and water, under 12 hour light/dark cycle and temperature of 22°C^±^2°C.

### Surgical procedures

The animals received anesthesia with xylazine (10 mg/kg - Anasedan, Agribrands do Brazil Ltda., Paulínia, SP, Brazil) and ketamine (80 mg/kg - Dopalen, Sespo Ind. & Com. Ltda., Jacareí, SP, Brazil), they had their fur of dorsal region shaved and antisepsis was performed with 5% iodine solution. In total, 30 rats were divided into five analysis periods (six rats per period: 7,15,30,60, and 90 days) being randomly selected using a computer-generated table. Polyethylene tubes were implanted according to previous studies.^5,14,16,17,19-21^

The animals received intravenous dypirone (0.03 mg *per* 100 g of body weight) during the first three days. Also, rats’ feeding and drinking pattern and possible changes in behavioral profile were assessed. After 7, 15, 30, 60, and 90 days since implantation, the animals were euthanized with a lethal dose (150 mg/kg body weight) of sodium thiopental (Thiopentax^®^, Cristalia Produtos Quimicos Farmaceuticos Ltda., São Paulo, SP, Brazil) and the tubes with adjacent tissues were removed and fixed in 10% formalin at pH 7.0 and processed with paraffin or glycol methacrylate. The blocks included in glycol methacrylate were transversely sectioned in 3-μm sections and stained with hematoxylin-eosin^19,20^and 10-μm sections were stained according to the von Kossa technique or remained unstained to be observed under polarized light.^19,20^ The blocks included in paraffin were sectioned in 5 μm for analysis of immunolabeling of IL-1β and TNF-α.

### Inflammatory tissue response

The average number of inflammatory cells (IC) was evaluated where the tissue had contact with the material at the uncovered part of the tubes on days 7, 15, 30, 60, and 90. A light microscope (DM 4000 B; Leica, Wetzlar, Germany) and an image analysis system (Image Pro-Express 6.0, Olympus) were used for analyses. For each specimen, three localities were selected at intervals of at least 100 μm and stained with hematoxylin and eosin; in each locality, a standardized field of 0.09 mm^2^ of the connective tissue totaling 0.27 mm^2^ per animal. In each area, the entire amount of IC was calculated applying the image analysis system at 400X magnification; in each specimen, the results were divided by the total area, and then, number of IC/mm^2^ was achieved.^5^ Regarding the fibrous capsules, they were considered thin if < 150 μm and thick if ≥ 150 μm.^14,19,20^

### Mineralization capacity

The mineralization assessment using the von Kossa method and polarized light was determined by the area marked in μm^2^ using Qwin software (Leica Microsystems, Wetzlar, Germany).

### Immunolabeling of IL-1β and TNF-α

The histological sections were submitted to indirect immunoperoxidase technique applying primary antibodies (1:100): IL-1β (SC-10593, Santa Cruz Biotechnology, Santa Cruz, CA, EUA) and TNF-α (SC-18319, Santa Cruz, CA, EUA) as previously described.^21,22^ A biotinylated secondary antibody was applied for 2 h and subsequently treated with conjugated streptavidin - HRP for 1 h (Universal Dako Labeled HRP Streptavidin-Biotin Kit^®^, Dako Laboratories, Carpinteria, CA, USA). Finally, 3,3’-diaminobenzidine tetrahydrochloride (DAB chromogen Kit, Dako Laboratories) was used as a chromogen. Specimens without primary antibody aforementioned were used as negative control.

A blinded certified histologist (E.E.) performed the immunolabeling analyses of TNFα and IL-1β in a semiquantitative way. Three histologic slices were considered for each animal. Positive immunoreactivity (IR) was defined as a brownish color in the cells’ cytoplasm and extracellular matrix. For immunolabeling of both cells and extracellular matrix are greatly relevant for the study, a semiquantitative analysis was performed, providing information about the number of immunoreactive cells and immunolabeling intensity of the extracellular matrix. The scores ranged between 1 and 4, where: 1 = absence of immunoreactive cells; 2 = low IR (a few immunoreactive cells and weak labeling of the extracellular matrix; around one-quarter of the immunoreactive cells); 3 = moderate IR (a fair quantity of immunoreactive cells and moderate labeling of the extracellular matrix; around half of the immunoreactive cells); and 4 = high IR (a vast quantity of immunoreactive cells and strong labeling of the extracellular matrix; around three-quarters of the immunoreactive cells).^23^

### Statistical analysis

All data were submitted to the Shapiro-Wilk normality test. Data of cell viability, histology (inflammatory cell number and mineralization capacity) were analyzed statistically by two-way ANOVA, followed by the Bonferroni correction. The Kruskal-Wallis followed by the Dunn’s test was used for immunohistochemical analyses. All analyses were carried out using the Graph Pad Prism (version 5.0) software program with 5% significance level

## Results

### Cytotoxicity

**Figure 1:**
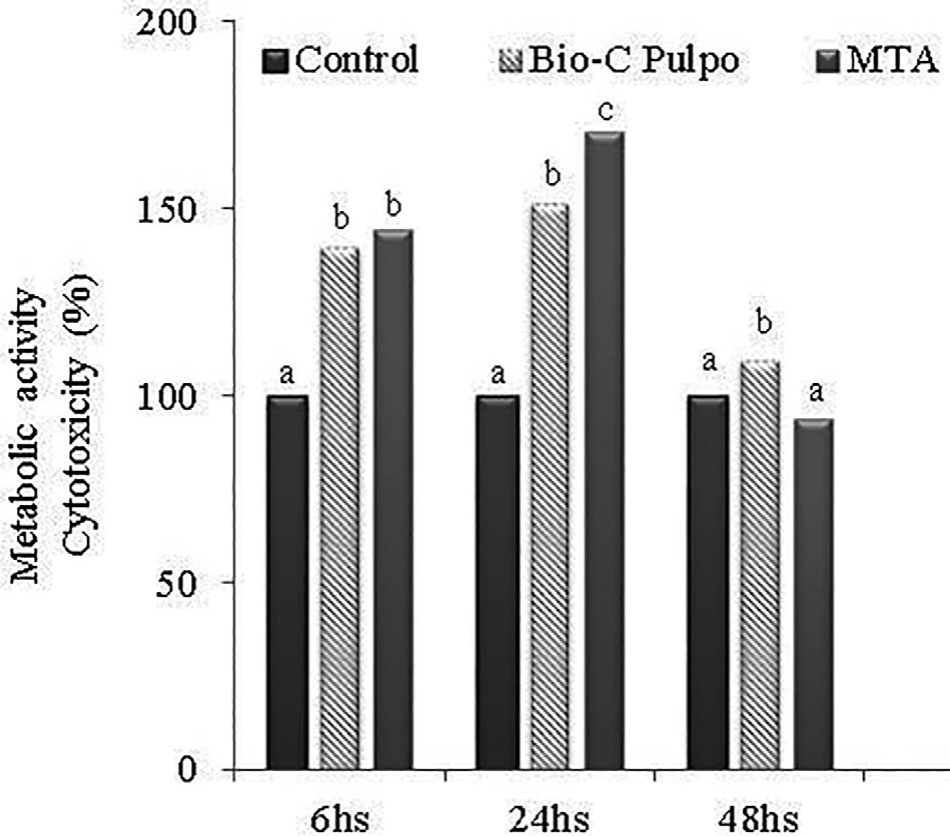
Cell viability of L929 fibroblasts after exposure with white MTA-Ang and BIO-C PULPO extract for 6, 24, and 48 hours determined by MTT assay. Note that white MTA-Ang and BIO-C PULPO presented higher cell viability when compared to the control group with decline in 48 hours in both materials. Different letters represent statistical differences between MTA and BIO-C PULPO (p<0.05).

The data of cytotoxicity of white MTA-Ang and BIO-C PULPO extracts are shown in Figure 1. The lowest percentage of cytotoxicity was found in the initial periods of analysis, declining in 48 hours period in both materials. At 6, 24, and 48 hours, white MTA-Ang and BIO-C PULPO presented higher cytocompatibility compared to the control group (p<0.05), except in 48 hours where white MTA-Ang was similar to control (p>0.05). At 6 hours, no statistically significant difference was found between white MTA-Ang and BIO-C PULPO (p>0.05). In 24 hours, cytocompatibility was higher for white MTA-Ang compared to BIO-C PULPO (p<0.05), and at 48 hours, BIO-C PULPO presented cytocompatibility greater than white MTA-Ang (p<0.05).

### Histologic Analysis

#### Control (empty tubes)

**Figure 2:**
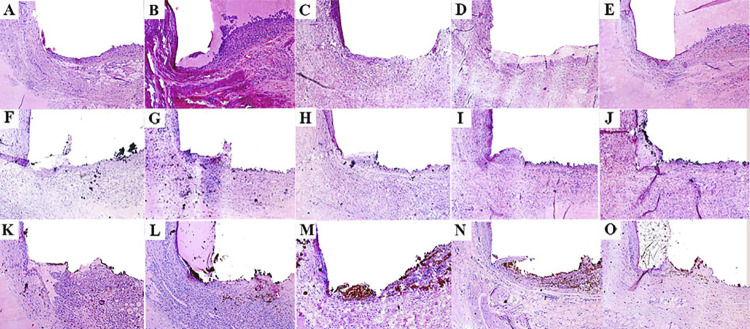
Representative images of the subcutaneous tissue reactions: Inflammatory response to the Control Group (empty tubes) at 7 (A), 15 (B) with intense inflammatory infiltrate and thick fibrous capsule; 30 (C), 60 (D) and 90 (E) days, with mild inflammatory infiltrate and thin fibrous capsule. White MTA-Ang at 7 (F), 15 (G) with moderate inflammatory infiltrate and presence of thick fibrous capsule; 30 (H), 60 (I), and 90 (J) days with mild inflammatory infiltrate and thin fibrous capsule; BIO-C PULPO at 7 (K), 15 (L), 30 (M) intense inflammatory infiltrate, thick fibrous capsule; 60 (N) and 90 (O) days with mild inflammatory infiltrate and thin fibrous capsule. Staining with hematoxylin-eosin shows fibrous capsule formation with infiltration of macrophages and lymphocytes (Scale bars: 200 μm; Original magnification: 100×).

**Figure 3:**
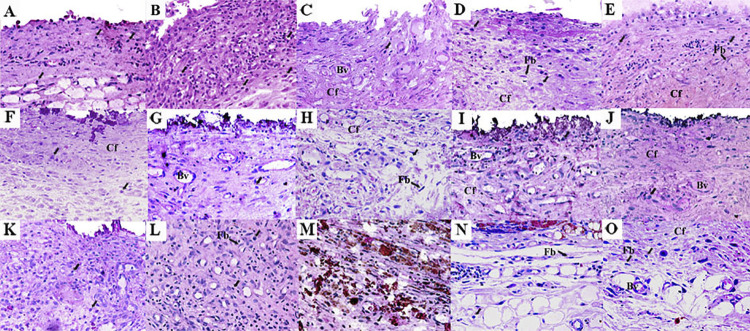
Representative images of the subcutaneous tissue reactions in the largest magnification: Control Group (empty tubes) at 7 (A), 15 (B), 30 (C), 60 (D) and 90 (E) days; White MTA-Ang at 7 (F), 15 (G), 30 (H), 60 (I) and 90 (J) days; BIO-C PULPO at 7 (K), 15 (L), 30 (M), 60 (N) and 90 (O) days. Several inflammatory cells (IC) (arrows) are observed, mainly in the portion of the capsule adjacent to the opening of the tubes. Typical fibroblasts (FB) are observed among bundles of collagen fibers (CF) in the capsules; blood vessels (BV). Haematoxylin-eosin (scale bars: 50 μm; original magnification: 400×).

**Figure 4:**
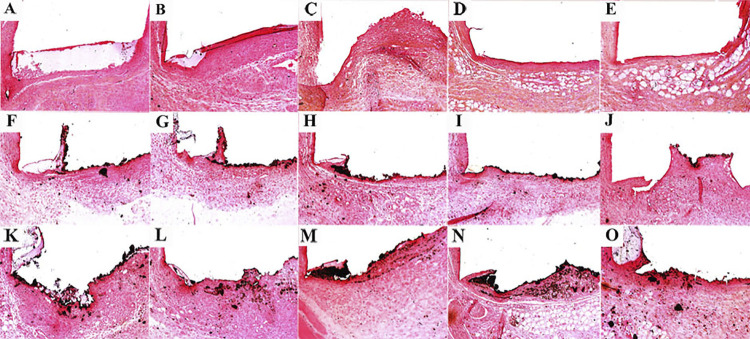
Representative images of the subcutaneous tissue reactions of mineralization in response to Control (empty tubes) at 7 (A), 15 (B), 30 (C), 60 (D) and 90 (E) days; white MTA-Ang at 7 (F), 15 (G), 30 (H), 60 (I) and 90 (J) days and BIO-C PULPO at 7 (K), 15 (L), 30 (M), 60 N) and 90 (O) days. Black areas represent mineralization from von Kossa method. Both materials induced mineralization, which was not observed in the control group (Scale bars: 200 μm; Original magnification: 100×).

**Figure 5:**
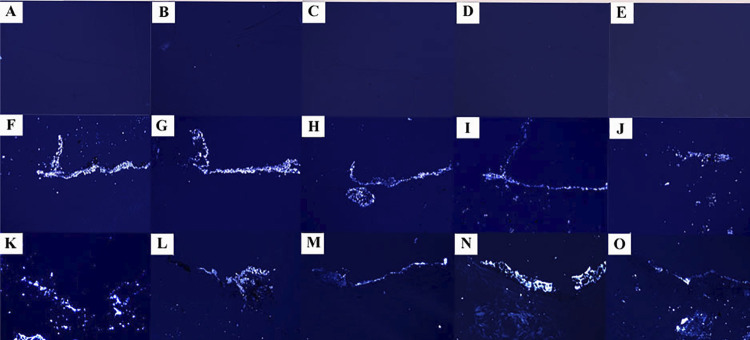
Representative images of the subcutaneous tissue reactions of mineralization in response to Control (empty tubes) 7 (A), 15 (B), 30 (C), 60 (D) and 90 (E) days; white MTA-Ang at 7 (F), 15 (G), 30 (H), 60 (I) and 90 (J) days and BIO-C PULPO at 7 (K), 15 (L), 30 (M), 60 (N) and 90 (O) days. Birefringent structures in the tissue represent calcite crystals, which were observed for both materials, but not in the control group (Scale bars: 200 μm; Original magnification: 100×)

On days 7 and 15, an intense inflammatory infiltrate was observed with thick fibrous capsule on the tube opening (Figure 2 and 3: A and B). The intensity of the inflammatory infiltrate was reduced on days 30, 60, and 90, presenting thin fibrous capsule (Figure 2 and 3: C-E). Significant difference was only observed when day 7 results were compared to days 30, 60, and 90 with a higher average number of inflammatory cells on the day 7 (p<0.05) (Table 1). No positive reaction was observed for von Kossa (Table 1) (Figure 4: A-E) and birefringent structures under polarized light (Table 1) (Figure 5: A-E).

**Table 1:** Number of inflammatory cells, area of mineralization by von Kossa (VK) (μm2), area of mineralization by polarized light (POL) (μm2), thickness of fibrous capsule and scores of immunolabeling for IL-1β and TNF-α

Time	Material	Inflammatory Cells	VK	POL	Fibrous capsule	Immunolabeling	
		Mean ±SD*	Median (min-max values)^**†**^	Median (min-max values)^**†**^		Scores (median)^**§**^ IL-1β TNF-α	
7 days	Control	299 (±29)^aA^	0 (0-0)^aA^	0 (0-0)^aA^	Thick	2^a^	2.5^a^
	MTA	214 (±50)^bA^	51,365 (31,723-99,329)^bA^	64,729 (38,748-89,105)^bA^	Thick	2^a^	2.5^a^
	BIO-C PULPO	328 (±69)^aAB^	15,538 (1,681-178,434)^bA^	39,930 (2,980-128,260)^abA^	Thick	3^a^	3^a^
15 days	Control	254 (±65)^aAB^	0 (0-0)^aA^	0 (0-0)^aA^	Thick	2^a^	2^a^
	MTA	212 (±45)^aA^	51,253 (32,558-73,778)^bA^	45,671 (24,169-96,900)^bA^	Thick	2^a^	2^a^
	BIO-C PULPO	396 (±84)^bA^	24,142 (20,131-33,237)^abA^	24,446 (12,515-34,351)^abA^	Thick	2^a^	2.5^a^
30 days	Control	146 (±50)^aB^	0 (0-0)^aA^	0 (0-0)^aA^	Thin	2^a^	2^a^
	MTA	206 (±88)^bA^	27,652 (20,184-32,611)^bB^	29,127 (17,423-43,998)^bAB^	Thin	2^a^	2^a^
	BIO-C PULPO	294 (±107)^bAB^	35,387 (25,120-999,70)^bA^	21,538 (11,738-34,472)^bA^	Thin	2^a^	2^a^
60 days	Control	134 (±21)^aB^	0 (0-0)^aA^	0 (0-0)^aA^	Thin	2^a^	2^a^
	MTA	157 (±64)^aA^	24,965 (16,840-31,919)^abAB^	24,836 (21,956-31,085)^bAB^	Thin	1.5^a^	2^a^
	BIO-C PULPO	204 (±57)^aBC^	44,193 (30,507-67,682)^bA^	29,396 (18,285-58,402)^bA^	Thin	1.5^a^	2^a^
90 days	Control	144 (±8)^aB^	0 (0-0)^aA^	0 (0-0)^aA^	Thin	1^a^	1^a^
	MTA	135 (±31)^aA^	16,046 (3,889-16,979)^bB^	15,458 (10,961-30,939)^bB^	Thin	1^a^	1^a^
	BIO-C PULPO	138 (±24)^aC^	27,992 (19,820-35,860)^cA^	16,897 (12,563-17,232)^bA^	Thin	1^a^	1^a^

*,^†^,^§^Different lowercase letters indicate statistical difference between groups at each time of analysis (p<0.05); different uppercase letters indicate statistical difference between each group at different analysis times (p<0.05).

*One-way ANOVA followed by Tukey’s test and ^†^Kruskal-Wallis test followed by Dunn’s test, after normality test (p<0.05).

^§^Kruskal-Wallis test followed by Dunn’s test (p<0.05).

#### White MTA-Ang

On days 7 and 15, a moderate inflammatory infiltrate with the presence of thick fibrous capsule was observed on the tube opening (Figure 2 and 3: F and G). The intensity of the inflammatory infiltrate was reduced on days 30, 60, and 90, presenting thin fibrous capsule (Figure 2 and 3: H-J). No significant difference was observed across the timepoints analyzed (p>0.05) (Table 1). Larger mineralization area in von Kossa was found on days 7 and 15 than the other timepoints (p<0.05) (Table 1) (Figure 4: F-J). Birefringent structures to polarized light on days 7 and 15 were present in a higher number compared to that observed on day 90 (p<0.05) (Table 1) (Figure 5: F-J).

## BIO-C PULPO

On days 7, 15, and 30 an intense inflammatory infiltrate was observed, it also presented a thick fibrous capsule. (Figure 2 and 3: K-M). On days 60 and 90, the inflammatory infiltrate was reduced, presenting a thin fibrous capsule (Figure 2 and 3: N and O). A higher mean number of inflammatory cells was observed on day 7 in comparison with day 90 (p<0.05); the same occurred on day 15 when compared to days 60 and 90 (p<0.05); and for the comparison between day 30 and day 90, the first period presented more intense inflammation (p<0.05) (Table 1). There was no significant difference for von Kossa and for birefringent structures to polarized light (Table 1), (Figure 4: K-O), (Figure 5: K-O), respectively.

## Comparison between the different groups not even time of analysis

### Inflammatory response

The average number of inflammatory cells in the tube was determined on days 7, 15, 30, 60, and 90 and data were compared at each timepoint (Table 1). On day 7, there was a significant difference between control and white MTA-Ang, as well as white MTA-Ang and BIO-C PULPO; with a higher average number of inflammatory cells for BIO-C PULPO (p<0.05). After 15 days, Control/white MTA-Ang and BIO-C PULPO were different, with a higher average number of inflammatory cells for BIO-C PULPO (p<0.05). On day 30, a statistical difference was observed between the control group and white MTA-Ang/BIO-C PULPO with the highest average of inflammatory cells for BIO-C PULPO (p<0.05), but there was no statistical difference between white MTA-Ang and BIO-C PULPO (p>0.05). After 60 and 90 days, there was no difference in the number of inflammatory cells among groups (p>0.05) (Table 1).

### Mineralization

Tissue sections of specimens underwent histochemical method for detecting calcification (von Kossa method). Regions of the capsule impregnated in black were found (positive to the method), except for the control group (Table 1). On days 7, 15, 30, and 60, no significant difference occurred between white MTA-Ang and BIO-C PULPO for von Kossa analyze (p>0.05). On day 90, greater area of mineralization in von Kossa was found for BIO-C PULPO compared to white MTA-Ang (p<0.05). Birefringence under polarized light, revealed the presence of calcium carbonate particles in all analyzed periods (p>0.05), excepting the control group. There was no statistically significant difference in the timepoints analyzed for birefringent structures under the polarized light (p>0.05).

## Immunohistochemistry

**Figure 6:**
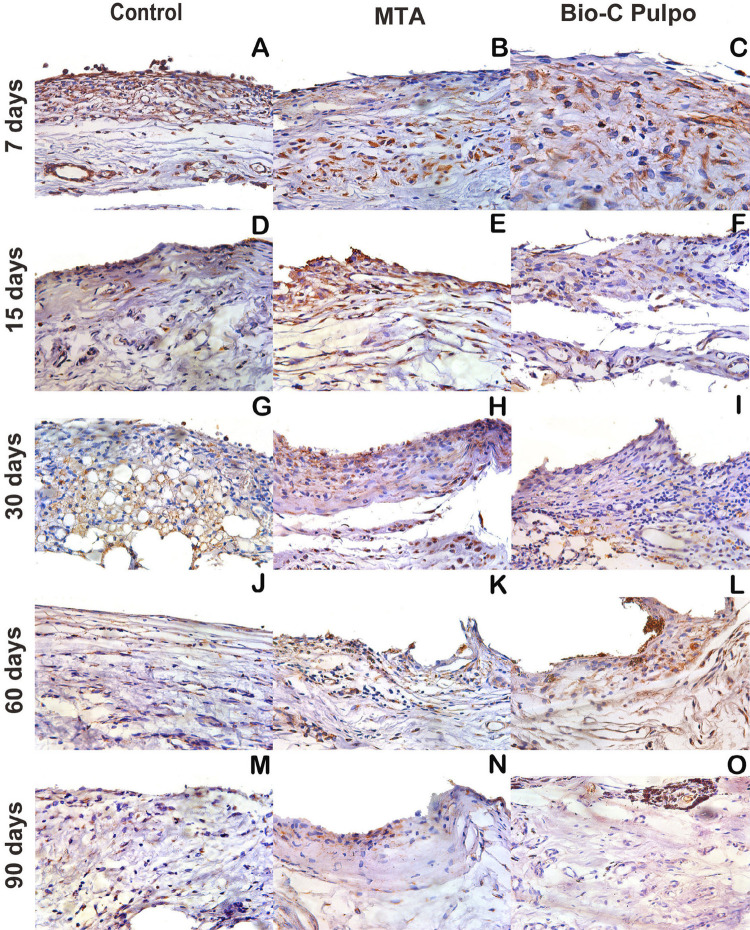
Photomicrographs showing the histological appearance representative of immunolabelling on Control, MTA and BIO-C PULPO for TNFα. Brownish colour presented predominantly in the cytosolic compartment of the cells and in the extracellular matrix. (A–O). Harris haematoxylin counterstaining. (scale bars: 100 μm; original magnification: 1000×)

**Figure 7:**
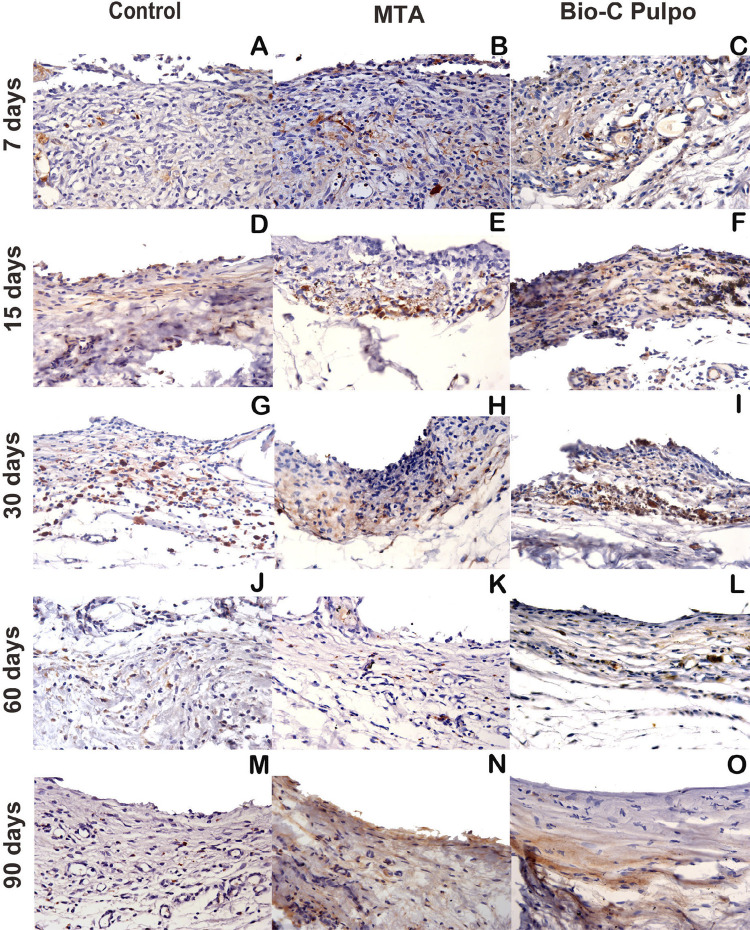
Photomicrographs showing the histological appearance representative of immunolabelling on Control, MTA and BIO-C Pulpo for IL-1β. Brownish colour presented predominantly in the cytosolic compartment of the cells and in the extracellular matrix. (A–O). Harris haematoxylin counterstaining. (scale bars: 100 μm; original magnification: 1000×)

The immunoreactivity patterns for IL-1β and TNF-α are showed in Table 1. Immunoreactive cells presented dark brown staining limited to the cytoplasm and, at a lesser intensity, in the extracellular matrix (Figures 6 and 7). No statistical differences were found between Control, BIO-C PULPO, and white MTA-Ang in the different periods of analysis (p>0.05).

## Discussion

Although a prior study exists evaluating the inflammation of BIO-C PUPO in rat subcutaneous tissue,^21^ this study complements the previous study by evaluating the cytotoxicity in fibroblasts, mineralization with presence of calcite crystals and immunolabeling for IL-1β and TNF-α. Several approaches are used to assess the biological behavior of endodontic materials. *In vitro* studies are the foremost assessing the biocompatibility of materials and may reflect their cytotoxicity in an isolated environment and mono cell layer, while *in vivo* studies represent the second level of analysis and they present the tissue response to a material that attempts to compensate some deficiencies of the *in vitro* model such as cell type, tissue organization, inflammatory reaction, immune reaction, among others.^14,24,25^ Thus, both methodologies were used to investigate BIO-C PULPO.

In this study, the L929 cell line, a lineage commonly used for cytotoxicity analyses of endodontic materials.^14,15,17^ was employed, aiming to evaluate cell viability by MTT assay. This variety of cells have some advantages over cells of primary lineages, such as the ability of constant growth whenever favorable conditions for it exists; differently from cells of the primary lineage, which die as soon as they reach growth plateau.^14^ Furthermore, these cells were selected for the materials tested would be in contact with *in vivo* fibroblasts.^14,17^

Despite a recent study indicating to the initial setting time of the BIO-C PULPO as approximately 7 min,^10^ the final setting time of this material has not yet been evaluated. Meanwhile, there is a lack of consensus on the final setting point of white MTA-Ang.^26^Thus, we chose to maintain 6 hours for setting the materials, as other studies that evaluated the cytotoxicity of white MTA-Ang.^14,16^Also, as the original extract of the white MTA has already been shown as non-cytotoxic in other studies,^26,14,16^we chose to compare the cytotoxicity of the original BIO-C PULPO extract, without using their dilutions. ISO also allow and recommend the use of the original extract.^15^

It was observed that BIO-C PULPO and white MTA-Ang presented cytocompatibility at 6, 24, and 48 similar or higher than the control for all evaluated periods. This result may occur due to calcium silicate being the main component of BIO-C PULPO, and this component is associated with the preservation of cellular viability, as well as increasing cell proliferation.^27^ Some differences between the materials can be related to radiopacifier, once BIO-C PULPO has calcium tungstate and white MTA-Ang has bismuth oxide. The presence of bismuth oxide radiopacifier in the composition of white MTA-Ang has been associated with higher cytotoxicity.^28^ Also, BIO-C PULPO contains calcium hydroxide which is related to maintenance of cell viability and also promotes conditions for cells’ proliferation.^6,14^Regarding cytotoxicity BIO-C Pulpo was more reactive than MTA in 24 and 48 hours. A recent study showed that cytotoxicity of BIO-CPULPO in 24 hours may be related to aggressive leaching occurring during the setting reaction.^10^ Furthermore, in the most advanced periods (i.g 48 hours) cytotoxicity may be higher due to the saturation of hydroxyl ions.^10^Moreover, BIO-C PULPO had the highest calcium release when compared to other cements, such as Biodentine, TotalFill Root Repair Material (FKG, Brasseler, Savannah, USA) and Theracal (Bisco, Inc., Schaumburg, IL, USA). This may have occurred due to the incorporation of silicon oxide in the composition of BIO-C PULPO. Silicon oxide is included to convert calcium hydroxide into long-term calcium silicate hydrate to strengthen the material.^10^

Subcutaneous implantation was used for *in vivo* analysis of the tissue response to BIO-C PULPO and white MTA-Ang including inflammatory reaction and mineralization capacity.^5,20,29,30^This study showed that BIO-C PULPO and white MTA-Ang led to more intense inflammatory reaction on days 7 and 15 than at other periods of analysis, which can be explained by the inflammation originated with the implanted materials or by surgical procedures itself.^31^ However, if the inflammatory process persists for a longer period of observation, it can be attributed to the material properties, rather than to traumatic event of the tube implantation.^32^ On the other hand, after 30 days of experimentation, no difference was presented in this study in the average number of inflammatory cells between white MTA-Ang and BIO-C PULPO. The adequate reaction observed with BIO-C PULPO may occur due to its bioceramic composition.

In another study,^21^ BIO-C PULPO and white MTA-Ang induced a similar inflammatory response in all periods. However, in this other study, inflammation analysis was performed by scores, and paraffin was used as the method of including the specimens, which differs from our study, that used quantitative analysis of the inflammatory cells as well as using the resin as a material for inclusion. Resin (glycol methacrylate) embedding for the histological process presents some good aspects: preservation of cell morphology, enabling the definition of inflammatory process degree.^24^ Probably, the difference in the evaluation of inflammatory process between these studies may be due to both method of evaluation and inclusion method. However, considering the difficulty to perform immunohistochemical in resin slices, paraffin was also used to get around this negative point.

The immunolabeling of IL-1β and TNF-α for BIO-C PULPO and white MTA-Ang was similar in all analyzed periods. Pro-inflammatory cytokines are generated mainly by activated macrophages and they perform a significant role in the up-regulation of inflammatory reactions.^33^ TNFα acts in the response to tissue damage or infection by evoking inflammation, recruiting lymphocytes and monocytes to the infection sites, and stimulating endothelial cells to display adhesion molecules and release chemokines.^34^ The cytokine protein IL-1β is responsible for enrolling the inflammatory cells by inducing adhesion molecules on endothelial cells and the release of chemokines by stromal cells. Furthermore, phospholipase A2, cyclo-oxygenase 2, and inducible nitric oxide (NO) synthase are also produced by it, driving the release of prostaglandin E2 and NO, important inflammatory mediators that contribute to local and systemic responses.^35^ Despite the differences in composition, BIO-C PULPO and MTA presented similar immunolabeling, which suggests an equivalent efficacy between cements.

The BIO-C PULPO has components that promote adequate tissue response. The zirconium oxide was used as a radiopacifier in BIO-C PULPO once bismuth oxide has drawbacks related to the discoloration, which can be attributed to its interaction with the collagen present in tooth tissue. It has been established that zirconia oxide used as radiopacifier did not interact with collagen and may prevent tooth discoloration, which reinforce the hypothesis that bismuth oxide is associated with MTA darkening even in the white formulation.^13^ Besides, zirconium oxide causes a lower inflammatory reaction in tissues compared to bismuth oxide.^36^

Moreover, the manufacturer states that silicon dioxide was added to bring viscosity to the material, thus improving its insertion into the cavity. Furthermore, distilled water was replaced with a liquid containing distilled water, plasticizing material, calcium chloride, and methylparaben. The plasticizer provides higher plasticity, improving material handling and insertion into cavity. However, the manufacturer did not disclose the plasticizer composition. Calcium chloride was added to provide shorter setting time,^37^ iron oxide was added to act as pigmentation agent, similar to that found in MTA^7^ and methylparaben was inserted as a preservative.^38^

Calcium fluoride was inserted in BIO-C PULPO. The endodontic materials that contain fluoride compounds have an improved penetrability into dentin and they also present increased mineralization.^39^ Specifically, the addition of calcium fluoride improves the ability of forming apatite.^40^Once BIO-C PULPO is a bioceramic, it was expected that positive von Kossa and also birefringent structures in polarized light were similar or superior to the white MTA-Ang. It was confirmed by a larger area in the BIO-C PULPO group for von Kossa on day 90, which was significantly different compared to the white MTA-Ang, thus suggesting a longer time for calcium carbonate formation, possibly due to its formulation.

Calcium silicate together with calcium fluoride, calcium aluminate, and calcium hydroxide promote deposition of mineralized structures in BIO-C PULPO. In calcium silicate-based material bioactivity occurs after hydration.^25,41^ The contact of materials with tissue triggers the reaction of Ca^2^ ions with OH^−^ ions derived from water, forming the calcium hydroxide, that creates a highly alkaline environment.^37^ The calcium ions react with carbon dioxide present in the tissues, originating calcite crystals and a reduced inflammatory process.^25^ It has been suggested that these crystals originated from the precipitation of calcium carbonate could stimulate the deposition of mineralized tissue detected by von Kossa and also birefringent structures in polarized light.^41^ Also, these calcified structures in subcutaneous investigations are a sign of osteoinductivity of the tested material.^5,20,27,41^

This type of study is the first stage of research on the use of new endodontic materials and, therefore, it should be interpreted with caution. However, its execution is greatly relevant since the use of cells and animals enable work in a highly controlled environment, focusing only on variables necessary for a preliminary understanding of the material response during future clinical use.

## Conclusion

BIO-C PULPO presents similar results to the control in the metabolic activity of cell and it induces tissue reaction similar to white MTA-Ang including stimulation of mineralized tissue formation. Therefore, BIO-C PULPO was considered a biocompatible material.
